# Short Stay Thyroid Surgery: Can We Replicate the Same in Low Resource Setting?

**DOI:** 10.1155/2018/4910961

**Published:** 2018-08-05

**Authors:** Naval Bansal, Sanjay Kumar Yadav, Saroj Kanta Mishra, Kamal Kishore, Anjali Mishra, Gyan Chand, Gaurav Agarwal, Amit Agarwal, Ashok Kumar Verma

**Affiliations:** ^1^Consultant Breast and Endocrine Surgeon, Fortis Healthcare, Mohali, India; ^2^Senior Resident, Department of Endocrine Surgery, Sanjay Gandhi Postgraduate Institute of Medical Sciences, Raebareli Road, Lucknow 226 014, India; ^3^Professor, Department of Endocrine Surgery, Sanjay Gandhi Postgraduate Institute of Medical Sciences, Raebareli Road, Lucknow 226 014, India; ^4^Provisional Fellow, Department of Anaesthesia and Perioperative Medicine, Royal Hobart Hospital, Hobart, Tasmania 7001, Australia; ^5^Additional Professor, Department of Endocrine Surgery, Sanjay Gandhi Postgraduate Institute of Medical Sciences, Raebareli Road, Lucknow 226 014, India

## Abstract

**Introduction:**

The concept of short stay thyroidectomy has been tested and in practice in the developed world; the same has not been replicated in countries with limited resources due to lack of organized healthcare system. So, in this study, we tried to analyze if short stay thyroid surgery can be performed in a cost-effective way in developing countries and also if the endocrine surgical trainee can deliver these services safely.

**Methods:**

The study was conducted prospectively from January 2013 to July 2014, at Department of Endocrine Surgery, SGPGIMS, Lucknow, India. Study group included patients undergoing short stay hemithyroidectomy whereas matched patients who qualified for inclusion criteria but did not undergo short stay surgery due to various reasons constituted control group. Outcome in both the groups was compared in terms of complication rates, cost benefit, and patient satisfaction. Subgroup analysis was also done for trainee versus consultant performed short stay thyroid surgery.

**Results:**

A total of 439 patients with surgical thyroid disorders were evaluated at our institute during the study period and out of these 110 patients (58 cases and 52 controls) fulfilled the inclusion criteria. Younger patients with low socioeconomic status who were paying out of pocket were found to be more inclined to short stay thyroid surgery. There was no significant difference between the two groups in terms of postanesthetic discharge score (PADS), complication rates, and patients satisfaction; however there was significant reduction (p <0.001) in hospital cost in short stay group. In subgroup analysis, procedure time was more in trainee performed surgeries; however there was no significant difference in terms of mean PADS and complication rates.

**Conclusion:**

Short stay thyroidectomy can provide a better cost-effective alternative to conventional thyroidectomy in patients undergoing thyroid surgery and can be safely performed by endocrine surgical trainees even in a low resource setting.

## 1. Introduction

In today's era with the advancement of technology and safe surgical techniques, “day care surgery” (admission, surgery, observation, and discharge on the same working day) and “short stay surgery” (surgery followed by overnight observation with discharge the following morning; typical stay duration of 23 h) are being undertaken for a growing list of surgical procedures including thyroid surgery [1, 2]. Though the concept has been tested and is in practice in the developed countries, the same has not been replicated in countries with limited resources [3, 4]. Day care/short stay thyroid surgery has been found to be as safe as in-patient thyroidectomy and also has added advantages in terms of reduced risk of infection, shorter convalescence time, high patient satisfaction, and low cost [5–7]. However, the majority of developing nations still lack adequate resources to practice day care/short stay thyroidectomy safely. Further, poor financial status of patients and lack of universal health insurance policy force patients to bear the expense of surgical care out of pocket and shortage of skilled surgeons and anesthesiologists adds to the gravity of the situation. While the economic and social satisfaction outcomes have been investigated in great length, competency of surgeons to practice the procedure has not yet been looked into [8]. So, in this study, we aim to analyze if short stay thyroid surgery can be replicated in low resource setting in a cost-effective way and also if the endocrine surgical trainees can deliver these services safely.

## 2. Methods

The study was conducted prospectively from January 2013 to July 2014, in the Department of Endocrine Surgery, Sanjay Gandhi Postgraduate Institute of Medical Sciences, Lucknow, India, a tertiary care academic institute. Study group included patients undergoing short stay thyroidectomy and control group included matched patients who qualified for inclusion criteria but did not undergo short stay surgery due to various reasons (refusal to consent for short stay surgery or sociologistic problems). Outcome in both groups was compared in terms of complication rates, cost benefit, and patient satisfaction. Subgroup analysis was also done for trainee versus consultant performed short stay thyroid surgery. Short stay was defined as surgery followed by overnight observation with discharge on the following morning; total hospital stay duration is less than 23 hours. Inclusion and exclusion criteria for the study group are listed in Table 1. Surgical procedures were performed under general anaesthesia with endotracheal intubation. Patients were injected with intravenous Midazolam, Fentanyl, and Propofol and were maintained on Vecuronium and Desflurane. Antiemetic in the form of Ondansetron was given in all patients before shifting the patient from operating room to the postoperative observation ward. All patients received antibiotic prophylaxis and for pain single dose of intravenous Paracetamol and thereafter oral ibuprofen for three days was prescribed. Thyroidectomy was conducted through cervicotomy approach in a standard way. Oral diet was allowed, as tolerated, during the immediate postoperative period. All patients were evaluated next day morning according to the Postanesthetic Discharge Scoring System (PADSS) [10] and were discharged if score was more than nine. On discharge, each patient was counseled and received written and verbal instructions for wound management, postoperative pain, and signs/symptoms associated with possible complications. Patients were followed up at two weeks, six weeks, and at six months following surgery to look for short and long term complications. Results were analyzed using SPSS version 17.0. Independent t test and Chi-square tests were used for statistical analysis.

## 3. Results

During the study period a total of 439 patients with surgical thyroid disorders were evaluated at our institute and 110 patients fulfilled the inclusion criteria. Out of these, 58 (52.73%) patients consented for short stay thyroid surgery and were included in study group whereas the remaining 52 (47.23%) were recruited in control group. Detailed clinical and socioeconomic profile is listed in Table 2.

There was significant difference in acceptance rate among the younger patients with low socioeconomic status. Also patients who were paying hospital bills out of pocket opted more for short stay thyroid surgery (p 0.036). However, there was no statistical significant difference between two groups in terms of mean distance from hospital, ASA grade, and thyroid volume.

There was no significant difference among both groups in terms of mean postanesthetic discharge score and complication rates (Table 3). In short stay group, one patient developed noncompressive hematoma within six hours of surgery which was managed conservatively and one had urinary retention in postoperative period. One postmenopausal female patient developed symptomatic hypocalcaemia and required calcium and vitamin D supplements. All three patients (5.17%) required extended stay for 48-72 hours. There was significant difference in terms of hospital stay and cost of treatment among both the groups; however, there was no significant difference in terms of patient's satisfaction. None of the patients required readmission. Eleven (18.97%) patients felt that they should have been kept for a longer duration (pain: 5, apprehension: 3, and seroma: 3 patients).

Among 58 short stay thyroid surgeries, endocrine surgery trainees performed 39 hemithyroidectomies whereas consultants performed 19 hemithyroidectomies. There was no significant difference in terms of mean thyroid volume in both the groups (Table 4). Procedure time was more in trainee performed surgeries (124.89±24.64 versus 108.77±30.79, p 0.051); however, there was no difference in terms of mean postanesthetic discharge score and complication rates in both the groups. In trainee performed subgroup, one patient had noncompressive hematoma and one developed hypocalcaemia.

## 4. Discussion

To the best of author's knowledge this study is unique in addressing safety, economics, and competency aspect of short stay thyroid surgery in a low resource scenario. As per the World Bank report, India belongs to lower middle income country group with 24.7% population living in extreme poverty [11, 12]. So, short stay surgeries can provide a more realistic approach for cost-effective healthcare if it can be achieved with reasonable outcome. In our study we found that patients who belong to low and middle income group were more inclined for short stay thyroidectomy. Similarly, short stay thyroidectomy was more often opted by patients paying hospital expenses out of pocket, so this subset of patients should be the initial target population for offering short stay surgery in developing countries.

Short stay thyroidectomy appears to be more feasible than day care thyroidectomy in a developing country like India as patients travel a longer distance due to availability of only a few specialized centers and lack of primary care physician unlike developed countries. Contrary to American Thyroid Association (ATA) guideline on outpatient thyroidectomy, in our study mean distance was 210.59±157.52Km, signifying that distance should not be kept as criterion in developing countries if we are offering short stay services as compared to outpatient thyroidectomy [8]. Postoperative hemorrhage is the single biggest concern in outpatient thyroid surgery with incidence of 0.9 to 2.1% [11]. One of our patients (1.73%) had noncompressive hematoma within six hours of surgery and was managed conservatively and did not require reoperation. More than 50% postoperative hemorrhages occur after six hours of surgery and studies have shown that postthyroidectomy bleed related mortality could be prevented by keeping the patients under observation for a little longer period [13, 14]. Hence, we feel that short stay in contrast to day care would be preferable in low resource scenario. One patient had temporary hoarseness of voice which recovered within three months without any intervention. Six patients developed seroma; out of these, three patients required aspirations. Both groups had similar level of satisfaction in terms of length of hospital stay; however pain and apprehension were two major factors decreasing the satisfaction level among study group. Proper counseling and other modalities of pain relief like cervical plexus block could have improved the satisfaction level [15].

Systemic studies have been conducted in various surgical specialties and they have compared the outcome of trainee performed surgeries; in terms of increased operating time, increased reoperation and readmissions rates [16–18] and similarly complication rates of thyroidectomy are more if performed by unskilled surgeons or surgeons with little experience [19–21]. Studies have shown that longer procedure time is associated with increased perioperative morbidity [6, 22]. In our study trainees took more time for hemithyroidectomy; however there was no difference in terms of postanesthetic discharge score and wound related morbidity. One patient in study group had acute urinary retention and one had dilutional hypocalcaemia that could be attributed to longer procedure time; however it was statistically nonsignificant.

Internationally, an income of less than $1.25 per day per head of purchasing power parity is defined as extreme poverty and by this estimate about 24.7 % of Indians live in extreme poverty [10, 11]. Average cost of in-patient hemithyroidectomy in matched sample at our hospital is $220; so an average sum of $60 (more than monthly income of 24.7% population) was saved per patient while maintaining safety and efficacy of surgical procedure, proving cost effectiveness of this approach.

This study has reported experience from a developing world. We preferred to restrict ourselves only to hemithyroidectomy in contrast to the other thyroid surgical procedures performed on short stay basis in developed countries in view of limited resources in public healthcare to attend to emergencies and thus compromising safety of the patients. However, with large experience, improved community healthcare support, and adoption of telehomecare, other thyroid surgical procedures can be considered for short stay surgery in due course of time.

## 5. Conclusion

Short stay thyroidectomy can provide a better cost-effective alternative to conventional in-patient thyroid surgery and can be safely performed by endocrine surgery trainees even in a low resource setting.

## Figures and Tables

**Table 1:** Inclusion and exclusion criteria [9].

Inclusion criteria	Exclusion criteria
Clinical	(i) Previous thyroid surgery
(i) Patients of all age group	(ii) Ultrasound-estimated thyroid volumes >80 ml
(ii) Patients of ASA grades I & II	(iii) Patients on drugs that have an effect on coagulation
(iii) First neck surgery	
(iv) Euthyroidism	
(v) Ultrasound estimated volume <80 ml	
(vi) Patients undergoing hemithyroidectomy	
Social-logistic	
(i) Established autonomy at admission	
(ii) Adequate home support at discharge	
(iii) Able to communicate	

**Table 2:** Patients clinical and socioeconomic profile.

Items	Study group (n=58)	Control group (n=52)	p value
Mean Age (in years)	31.66±10.15	37.60±13.51	0.010
Sex (M:F)	1:4.3	1:2.3	0.151
Financial status*∗*			
(i) Low income	3 (5.17%)	0	0.001
(ii) Middle income	49 (84.48%)	31 (59.62%)	
(iii) High income	6 (10.34%)	21 (40.38%)	
Mode of payment			
(i) Self	53 (91.38%)	40 (76.92%)	0.036
(ii) Insurance	5 (8.72%)	12 (23.08%)	
Mean Distance (in Km)	210.59±157.52	231.58±201.04	0.541
Thyroid volume (in ml)	23.79±17.69	26.02±18.92	0.528
ASA grade			
I	47 (81.03%)	40 (76.92%)	0.597
II	11(18.97)	12 (23.08%)	

*∗* Based on OECD working paper (http://www.oecd.org/dev/44457738.pdf).

**Table 3:** Outcome analysis.

Items	Study group (n=58)	Control group (n=52)	p value
Mean PADS	9.79	9.71	0.325
Complications			
(i) Hemorrhage	1 (1.72%)	1 (1.92%)	
(ii) Hoarseness of voice			
(a) Transient	1 (1.72%)	2 (3.85%)	
(b) Permanent	0	0	0.513
(iii) Seroma/ wound infection	6 (10.34%)	4 (7.69%)	
(iv) Others	2 (3.45%)	0	
Total duration of hospital stay (in hours)	25.05±9.5	106.44±39.74	<0.001
Cost of treatment (INR)	10409±1355	14041±4220	<0.001
Satisfaction level based on duration of hospital stay			
(i) Appropriate	47 (81.03%)	42 (80.77%)	0.972
(ii) Should have remained longer	11 (18.97%)	10 (19.23%)	
Final Histopathology			
(i) Benign	52 (89.65%)	45 (86.54%)	0.613
(ii) Malignant	6 (10.35%)	7 (13.46%)	

**Table 4:** Trainee versus consultant performed short stay thyroidectomy.

Items	Trainee performed (n=39)	Consultant performed (n=19)	p value
Median thyroid volume (in ml)	25.66 ± 18.48	20.05 ± 15.79	0.263
Procedure time (in minutes)	124.89 ± 24.64	108.77 ± 30.79	0.051
PADS	9.79 ± 0.409	9.79 ± 0.419	0.963
Complications			
(i) Hemorrhage	1 (2.56%)	0	0.686
(ii) Hoarseness of voice			
(a) Transient	0	1 (5.26%)	
(b) Permanent	0	0	
(iii) Seroma / wound infection	3 (7.69%)	3 (15.79%)	
(iv) Others	2 (5.13%)	0	
Final Histopathology			
(i) Benign	35 (89.74%)	17 (89.47%)	0.975
(ii) Malignant	4 (10.26%)	2 (10.53%)	

## Data Availability

The data used to support the findings of this study are available from the corresponding author upon request.
